# Supplementary material for: Conflict driven displacement and child health: Evidence based on mother’s nationality from Jordan Population and Family Health Survey
Source: PLoS One. 2021 Sep 7;16(9):e0257080. doi: 10.1371/journal.pone.0257080 (PMC8423276; doi:10.1371/journal.pone.0257080)
Supplement: S1 Table — (DOCX) [file pone.0257080.s001.docx]

**S1 Table Additional information of the variables that have been categorized in the data analysis.**

| **Outcome Variable** | Mothers Nationality (Jordanian==0), (Syrian==1) and (Others==2) |
| --- | --- |
|  |  |
| **Explanatory Variables** | |
|  |  |
| **Place of Residence** | (Urban==1) and (Rural==2) |
| **Mothers' Education** | No Education or illiterates (0) Primary up to 5^th^ standard (1) Secondary ( Up to 10^th^ (2) and Higher is Secondary and above (3) |
| **Wealth Index** | Poorest (0) Poorer (1), Middle (2), Richer (3) and Richest(4) |
| **Sex of the children** | (Male child =1) Female child (2) |
| **Age** |  |
| **Mothers Work status** | (Not Working = 1) (Working=2) |
| **Region** | Central (Irbid, Jarash, Ajloun, and Mafraq), ( Central (Amman, Zarqa, Balqa, and Madaba) and South (Karak, Tafiela, Ma’an, and Aqaba) |
| **Birth Order** | (Up to two Births==1) (Three to five births==2) (Six and above==3) |
| **Number of children living** | Single child ==1, Two to three children==2, and More than three==3 |
| **Mothers having health card** | (No=1) (Yes=1) |
| **Child received BCG** | (No==1) (Yes==1) |
| **Sources of water** | Piped water ==1 and Bottled water ==2 |
| **Plain Water** | (No=1) (Yes=1) |
| **Having any toilet Facilities** | No==1 and Yes==2 |
| **Fuel type** | Smokeless fuel ==1 and Smoking fuel == 2 |
| **Mother smoking cigarette** | (No=1) (Yes=1) |
| **Mother smoking** | (No=1) (Yes=1) |
