# Supplementary material for: Conflict driven displacement and child health: Evidence based on mother’s nationality from Jordan Population and Family Health Survey
Source: PLoS One. 2021 Sep 7;16(9):e0257080. doi: 10.1371/journal.pone.0257080 (PMC8423276; doi:10.1371/journal.pone.0257080)
Supplement: Attachment — Submitted filename: Response to Reviewers.docx [file pone.0257080.s002.docx]

**Title: Conflict driven displacement and child health inequality: Evidence based on mother’s nationality from Jordan Population and Family Health Survey**

Dear All,

We would like to thank you for these constructive, building and improvable comments on this manuscript that would improve the manuscript's substance and content. We considered each comments and clarification questions of the editor and reviewers on the manuscript thoroughly. Our point-by-point responses for each comment and questions are described in detailed on the following pages. Further, the details of changes were shown by track changes in the supplementary document attached. Once again, thank you for your valuable insights.

**Editor’s comments**

1. Please ensure that your manuscript meets PLOS ONE's style requirements, including those for file naming. The PLOS ONE style templates can be found at

<https://journals.plos.org/plosone/s/file?id=wjVg/PLOSOne_formatting_sample_main_body.pdf> and

<https://journals.plos.org/plosone/s/file?id=ba62/PLOSOne_formatting_sample_title_authors_affiliations.pdf>

*Authors’ response:* Dear Editor, Thank you for the suggestion. We have prepared the manuscript as per the PLOSONE’s style including file name.

2. In your Methods section, please provide additional information about the eligibility criteria applied in your analysis; and please clarify how every variable was categorised.

*Authors’ response:* Thank you. We have prepared the additional information about the outcome and explanatory variables used in the data analysis. This can be found in S1 Table. Kindly see the supporting information’s file.

3. Please amend your manuscript to include your abstract after the title page.

*Authors’ response:* Thank you for your valuable suggestion. We have included the abstract after the title page.

**Reviewer #1**

*Comment:* Useful literature can be taken up in the discussion section. These papers discuss the risk factors for unexpected child death, sudden infant death and stillbirth in Jordan and the many issues raised regarding the health care system, high smoking rate and smoking restrictions, as well as cultural, economic and risk groups.

*Authors’ response:* We have included literature as suggested by the reviewer in the discussion section. Thank you for the informative suggestion.

**Reviewer #2**

1. The discussion contradicts the result on diarrhea. The result shows that prevalence of diarrhea is higher in Jordanian children than Syrians and other nationalities.

*Authors’ response:* Thank you for your comments we have critically revised our discussion and made it consistent with the study findings in the revised draft, we kindly suggest you to go through the revised draft.

2. The difference in health outcomes seems correlated to socio-economic indictors than nationality. The authors should focus the article on the socio-economic disparity between refugees and Jordanians affecting health of children. The nationality argument not supported well.

*Authors’ response:* Thank you for highlighting the core issue of this paper. As we framed this study, we also have a similar concern, but our aim was to examine the possible health risk due to conflict-driven displacement despite having a good health system as in the case of Jordan, which has one of the best health care system accommodating millions of refugees in the world. So, we included nationality as an important factor in our study.

Secondly, since this was a study based on secondary data, without any direct evidence of the mother’s being likely from Syria. So, we derived a notion based on some earlier literature and theoretical construct to establish the link between conflict and child health outcomes via mother’s nationality.

Thirdly health outcomes had a strong correlation with the children’s nationality derived from their mother’s. However, socio-economic indicators were also correlated with child health outcomes, but since our theoretical notion was more about displacement, so that was the best proxy indicator available in the dataset to be undertaken. Furthermore, socio-economic disparities have been already examined as cited by our study, so we used nationality as a construct to study health outcomes.

Lastly, although, we could not find sufficient evidence to support the nationalities argument, except denoting it as proxy mainly derived from conflict-driven displacement. But we did mention it as a possible limitation in our study.

3. How does the ARI, diarrhea and fever data look like by nationality, when controlled for socio-economic variables?

*Authors’ response:* Thank you. The results were similar even after controlling them, we have already taken this into account while carrying our initial analysis, but since our goal was to study nationality as a likely factor based on the notion of conflict-driven displacement, so we used multinomial modelling to accommodate it, rather than control it as a potential predictor in the final analysis.

4. I suggest including that routine immunization is free for all children under 5 in Jordan. However, the pneumococcal conjugate vaccine (PCV) is not part of Jordan's routine immunization program. When it is introduced, it will be available for all children under 5, including refugees.

*Authors’ response:* Thank you for your suggestion. We have taken note of it in our revised manuscript.

**Reviewer #3**

1. It is a very important study on the health of the children under five having different nationalities, all residents of Jordan.

*Authors’ response:* Yes. It is.

2. Please modify the title according to the objective of the study.

*Authors’ response:* Since the focus was more on establishing a connotation of conflict with health outcomes, we felt it more catchy, but we have modified the title and made it more precise and problem-oriented.

3. The objective of the study needs to be precisely restructured and focused.

*Authors’ response:* Thank you. We have restructured it and made it more relevant with title and theoretical construct.

4. The Abstract has to be written properly and concisely.

*Authors’ response:* Thank you for the suggestion. The abstract has been concisely written. We would like you to see the revised manuscript.

 5. The Conclusion of the study has to be consistent with the study findings.

*Authors’ response:* Thank you for your comment. We have revised the conclusion and made it consistent with the study findings.

6. In Table 2, sample size has to be recalculated.

*Authors’ response:* Thank you for your comment, we have recalculated the Table and attached the same in the updated version. We kindly suggest to see the revised supplementary document.

| **Nationality** | **ARI** | **Diarrhea** | **Fever** | **Total Sample*** |
| --- | --- | --- | --- | --- |
| **Jordanian** | 5.76 | 9.8 | 13.01 | 8065 |
| **Syrian** | 6.45 | 8.76 | 13.57 | 1191 |
| **Other** | 10.13 | 8.49 | 13.01 | 368 |
| **Total** | 6.02 | 9.62 | 13.09 | 9623 |
| *Unweighted total sample of Children by mother’s nationality | | | | |
| *Source: Jordan Demographic and Health Surveys 2017-18.* | | | | |

7. Please take out the wordings like PhD Scholar, only the PhD will suffice. What about the credentials of other coauthors, please mention.

*Authors’ response:* Dear Reviewer, I have updated my personal details concisely. But the online submission system didn’t show the updated version.

And I am not able to edit the other coauthors credential because I am not getting such options to edit in the online submission system. But I have mentioned in the cover letter. Kindly see the cover letter.

8. The limitations of the study are nicely narrated.

*Authors’ response:* Thank you.

9. Please make necessary corrections of grammar and restructuring the sentences.

*Authors’ response:* Thank you for your kind suggestions. The necessary corrections of grammar and reconstruction of the sentences have been made clearly. We kindly request you to kindly see the revised manuscript.
