# Supplementary material for: Conflict driven displacement and child health: Evidence based on mother’s nationality from Jordan Population and Family Health Survey
Source: PLoS One. 2021 Sep 7;16(9):e0257080. doi: 10.1371/journal.pone.0257080 (PMC8423276; doi:10.1371/journal.pone.0257080)
Supplement: Attachment — Submitted filename: Response.docx [file pone.0257080.s003.docx]

**Comments**

**Reviewer #2**: The topic of study is still relevant and important. However, I was not able to discern the man public health message from the data and discussions. The revised manuscript is even more confusing than the previous version. The results are not described and discussed in a meaningful and coherent way that makes sense in terms of their public health relevance. I could not understand, what the authors were trying to tell us. For instance, "results showed that Jordanian children whose mother has a smoking habit and Syrian children's mothers who do not smoke cigarettes have high ARI prevalence. Hence, our results clearly show that disease risk is greater among the children of Syrian or any other nationality than the Jordanian." The authors should have picked a few of the results, which they thought were most relevant to support the title of the article. In its current form, I do not see this article contains coherent and, from public health perspective, relevant new information that merits publication in **PLOS**

**Response**

Thank you for your critical evaluation. We have revised once again our paper and made the changes as highlighted by you. We have tried our best this time to make the results coherent with the final objective and title of the research paper.

Now I would like to come to your specific comments

**Regarding the public health message of the paper.**

While conceptualizing the paper, we tried to address some of the key aspects that are key in terms from public health perspective only. So, our aim was to make a comprehensive analysis of how health care system of a particular country gets affected by uncertain demands that arise mainly from the conflict in its neighbouring states. Whether the influx of refugees overshadows the health care services for natives. And with risk of vulnerability in health care outcomes how does these public health challenges impact the health of refugee’s especially vulnerable population like children. Therefore, based on the children sample determined by mother’s nationality we used the JDHS to analyze the health outcomes among the under-five children in Jordan including native Jordanian children and other nationality children.

We felt that the paper critically analyses the impact of conflict in health settings particularly in terms of provision of healthcare services and inequality in terms of access to health care. We therefore felt this an in important significant public health issues undertaken by the PLOSone in earlier research, that is why we opted for **this journal**, and felt that it will reach the wider audience given its strong implications in terms of health care access and provisions in children.

**Regarding your results suggestion**

Thank you for highlighting that we have already corrected it in our final revision.

I have hoped this revision of paper suits well and address the concerns, which you have pointed out. We will be really pleased if there is any other issue that we need to address in this paper. We would love to correct it before making it suitable for this publication.

Regards

Authors
